# miR-22 inhibits tumor growth and metastasis by targeting ATP citrate lyase: evidence in osteosarcoma, prostate cancer, cervical cancer and lung cancer

**DOI:** 10.18632/oncotarget.10020

**Published:** 2016-06-14

**Authors:** Mei Xin, Zhiguang Qiao, Jing Li, Jianjun Liu, Shaoli Song, Xiaoping Zhao, Ping Miao, Tingting Tang, Lei Wang, Weichun Liu, Xiaodi Yang, Kerong Dai, Gang Huang

**Affiliations:** ^1^ Department of Nuclear Medicine, Renji Hospital, School of Medicine, Shanghai Jiao Tong University, Shanghai 200127, China; ^2^ Shanghai Key Laboratory of Orthopaedic Implants, Department of Orthopaedics, Ninth People's Hospital, School of Medicine, Shanghai Jiao Tong University, Shanghai 200011, China; ^3^ Bone and Joint Research Center, The First Affiliated Hospital, Frontier Institute of Science and Technology, Xi'an Jiaotong University, Xi'an 710061, China; ^4^ Department of Orthopaedics, Alpert Medical School/Rhode Island Hospital, Brown University, Providence, RI 02903, USA; ^5^ Department of Gynecology and Obstetrics, Renji Hospital, School of Medicine, Shanghai Jiao Tong University, Shanghai 200127, China; ^6^ Department of Anesthesiology, Zhongshan Hospital, School of Medicine, Fudan University, Shanghai 200032, China; ^7^ The Key Laboratory of Stem Cell Biology, Institute of Health Sciences, Shanghai Jiao Tong University School of Medicine (SJTUSM) & Shanghai Institutes for Biological Sciences (SIBS), Chinese Academy of Sciences (CAS), Shanghai 200031, China; ^8^ Institute of Health Sciences, Shanghai Jiao Tong University School of Medicine (SJTUSM) & Shanghai Institutes for Biological Sciences (SIBS), Chinese Academy of Sciences (CAS), Shanghai, 200031, China

**Keywords:** miR-22, ACLY, metabolism, tumor, therapy

## Abstract

MicroRNAs (miRNAs) are non-coding small RNAs that function as negative regulators of gene expression involving in the tumor biology. ATP citrate lyase (ACLY), a key enzyme initiating *de novo* lipid synthesis, has been found to be upregulated in cancer cells, and its inhibition causes suppressive effects in a variety of tumors. At present, although several ACLY inhibitors have been reported, the potential role of miRNAs in interfering ACLY still needs further clarification. Herein, four different types of tumor cells including osteosarcoma, prostate, cervical and lung cancers were adopted in our study, and we have demonstrated that miR-22 directly downregulated ACLY. Moreover, miR-22 was proved to attenuate cancer cell proliferation and invasion, as well as promote cell apoptosis via inhibiting ACLY. Additionally, we confirmed the higher ACLY protein levels and the lower miR-22 expressions in hundreds of clinical samples of the four primary tumors, and a negative correlation relationship between ACLY and miR-22 was clarified. Finally, in the four animal models, we found that along with the loss of the ACLY expression, the miR-22-treated mice developed rather smaller tumors, less probabilities of distant metastasis, and fairly longer survivals. *De novo* lipogenesis suppression triggered by miR-22-ACLY axis may contribute to the inhibition of tumor growth and metastasis. These findings provide unequivocal proofs that miR-22 is responsible for the posttranscriptional regulation of ACLY, which yields promising therapeutic effects in osteosarcoma, prostate, cervical and lung cancers.

## INTRODUCTION

Cancer is one of the most devastating diseases that threats global human public health and life quality. Of all the neoplasms, lung cancer has both the highest incidence and death rates in males, as well as the second leading cause of cancer deaths among females [1, 2]. Prostate cancer is the second most frequently diagnosed cancer that accounts for the fifth place of lethal cancerous disease in males worldwide [3]. Cervical cancer is the fourth most common cancer among women, with about 70% of the cases occurring in developing countries [2, 3]. Osteosarcoma, the most common primary malignant tumor of bone, is responsible for approximately 6% of childhood malignancies and the third most prevalent cause of cancer in adolescents [4–6]. Hence, it is a globally extreme urgency to develop effective approaches for treating these cancers.

The reprogramming of metabolic pathways, such as lipogenesis shifts, is one of the most important features of cancer [7]. In order to meet the increasing needs of the rapid cell division and propagation, demand for energy and macromolecules is increased. To achieve this goal, cancer cells undergo substantial metabolic modifications including the enhancements of *de novo* lipid synthesis [8], which in turn makes major impacts on the tumor development and progression [9, 10]. ATP citrate lyase (ACLY), the precursor of *de novo* lipogenesis, catalyzes cytosolic citrate into acetyl-CoA which fuels both fatty acid synthesis and the mevalonate pathway. Recently, more and more researches have been focusing on the prominent role of ACLY in cancer owing to its overwhelming metabolic activity [11] and deregulated protein expression [12–14]. And the inhibition of ACLY with genetic or pharmacologic strategies promotes apoptosis and differentiation, leading to the suppressive effect in various cancers [15–18].

Cancer is an epigenetic disease as well [19]. Typical epigenetic alterations include changes in DNA methylation, histone modifications and microRNA expression [20]. Current studies have usually focused on the genetic level of ACLY, whereas additional studies are required to clarify the epigenetic role in the metabolic regulation. MicroRNAs (miRNAs), a class of endogenous small non-coding RNAs, are negative regulators of translation that bind to the 3′-untranslated region (3′UTR) of their target mRNAs. Functional miRNAs are involved in a variety of biological tumor processes, such as cell proliferation, invasion, apoptosis and cellular metabolism [19]. Although a number of miRNAs have been found to modulate energy metabolism by controlling the key enzymes of metabolic pathways [21–23], the potential miRNAs in the regulation of ACLY have yet to be completely evaluated.

In this study, we investigated the four representative tumors, including osteosarcoma, prostate, cervical and lung cancers, and uncovered that ACLY is directly downregulated by miR-22, which has implications for the potential therapeutic disruption of tumor development and progression.

## RESULTS

### ACLY is a direct target of miR-22

**Figure 1:**
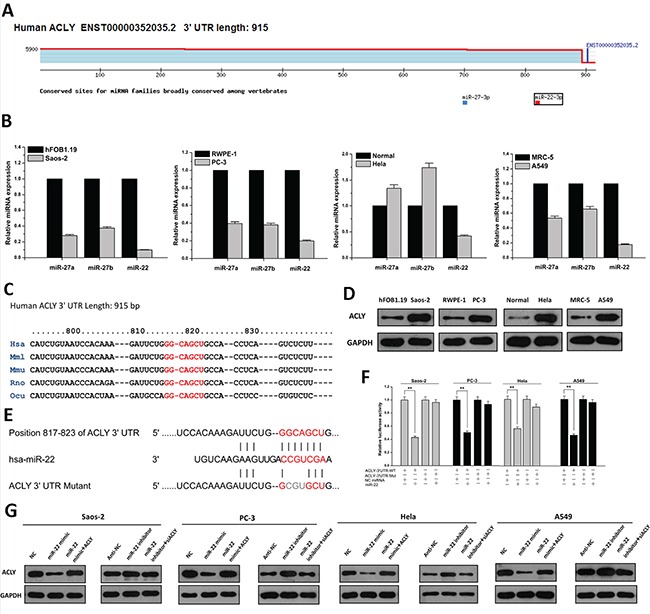
miR-22 directly targets ACLY **A.** Analysis of candidate miRNAs of ACLY conducted by Targetscan 7.0 software. **B.** Relative expressions of miRNA candidates quantified by real-time PCR in the Saos-2, PC-3, Hela, A549 cancer cell lines and their corresponding normal controls. **C.** A putative miR-22 binding site in the 3′UTR of ACLY mRNA across different species (shown in red). **D.** Differential expressions of ACLY protein levels in Saos-2, PC-3, Hela and A549 cells and their normal controls. **E.** Site-directed mutations of the miR-22 seeding region of ACLY 3′UTR (shown in grey). **F.** Relative luciferase activities of reporter plasmids of the four cancer cell lines cotransfected with psiCHECK-2-3′UTR-WT (ACLY-3′UTR-WT) or psiCHECK-2-3′UTR-Mut (ACLY-3′UTR-Mut), as well as NC miRNA or miR-22 mimic (**P<0.01). **G.** Analysis of ACLY protein expression in the four cancer cell lines treated with miR-22 mimic or miR-22 inhibitor as well as cotransfected by ACLY-overexpressed vector or siRNA.

To explore the probability of posttranscriptional control of ACLY regulated by miRNA, we used the bioinformatics tool TargetScan to predict the putative miRNA targeting ACLY. Although the 3′UTR of ACLY is relatively short, several miRNA candidates were found to bind different sites of the 3′UTR (Figure 1A). To identify the most optimal miRNA, quantitative real-time PCR was performed to compare the relative miRNA expressions between the cancer cells and the corresponding normal controls. As a result, miR-22, in particular, yielded a notable and consistent decline in the four tumor cells (Figure 1B). These findings indicated a potent functional connection between ACLY and miR-22, whose binding site was conserved across many other mammalian species (Figure 1C). On the other hand, we assessed the expression of ACLY protein in the cultured cells using western blots, and upregulated ACLY levels were uniformly found in the four malignant cells (Figures 1D and S1). Next, the wild-type 3′UTR of human ACLY mRNA was cloned into a luciferase reporter and site-directed mutagenesis was carried out to generate the mutant plasmid (Figure 1E). Compared to the NC miRNA, the luciferase activity of the wild-type reporter diminished by approximately 50% with the introduction of miR-22, and the mutant reporter was able to reverse this suppressive effect (*P*=0.000, separately; Figure 1F). To validate the downregulation effects of miR-22 in the tumor cell lines, western blots analyses against ACLY were used. Our results showed that in the Saos-2 cell lines, the ACLY protein levels dropped by around 50% after the miR-22 mimic transfection compared to the NC-treated cells, while the introduction of an overexpression plasmid could largely counteract the inhibitory effects that miR-22 exerted on ACLY. On the contrary, the miR-22 inhibitor enhanced the expression of ACLY, which restricted the interference of ACLY siRNA. Similar findings were confirmed in the other three cancer cells (Figures 1G and Supplementary Figure S1). All these results pointed out that ACLY is directly targeted by miR-22.

### miR-22 reduces *in vitro* cancer cell growth and invasion but promotes apoptosis by targeting ACLY

**Figure 2:**
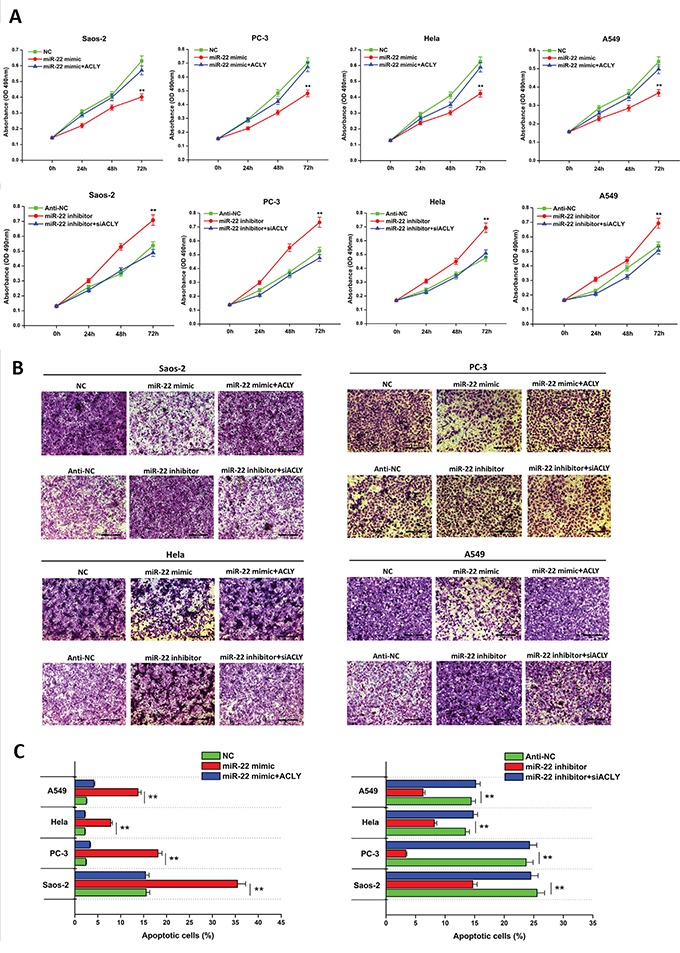
miR-22 attenuates cancer cell proliferation and invasion, but promotes cell apoptosis by targeting ACLY **A.** Cell proliferation analyses of the Saos-2, PC-3, Hela and A549 cells treated with the miR-22 mimic or inhibitor by the MTT assay (**P<0.01). **B.** Typical images of the cell invading behavior 24 hours after the transfection of the miR-22 mimic or inhibitor in the four tumor cells, respectively (100X, scale bar 200 μm). **C.** Analyses of the percentage of apoptotic cells in the four transfected cancer cell lines are displayed in the bar graphs separately (**P<0.01).

To evaluate the potential role of miR-22 in tumor biological processes by inhibiting ACLY, the four tumor cells were treated with the miR-22 mimic, miR-22 inhibitor, ACLY siRNA or the ACLY-overexpressed vector, and MTT assays were performed to detect cell proliferation. In the Saos-2 cells, for example, we found that the introduction of miR-22 led to an obvious defect in cell viability compared to the NC group, while the cell proliferating capacity could be partially compensated by the additional overexpression of ACLY. Parallel results were found in the other three cancer cells (Figure 2A). On the other hand, *in vitro* transwell assays showed that the treatment of miR-22 resulted in evident invasion suppressions in the relevant tumor cells compared with the NC-transfected ones (Figures 2B and Supplementary Figure S2). Besides, with the application of Annexin V/PI double-staining and the flowcytometry, we examined the sensitization of cell apoptosis influenced by miR-22. As illustrated in Figures 2C and S2, the treatments with the miR-22 accelerated the tumor cells towards apoptosis in comparison with the NC group.

### ACLY and miR-22 expression in primary tumors

**Figure 3:**
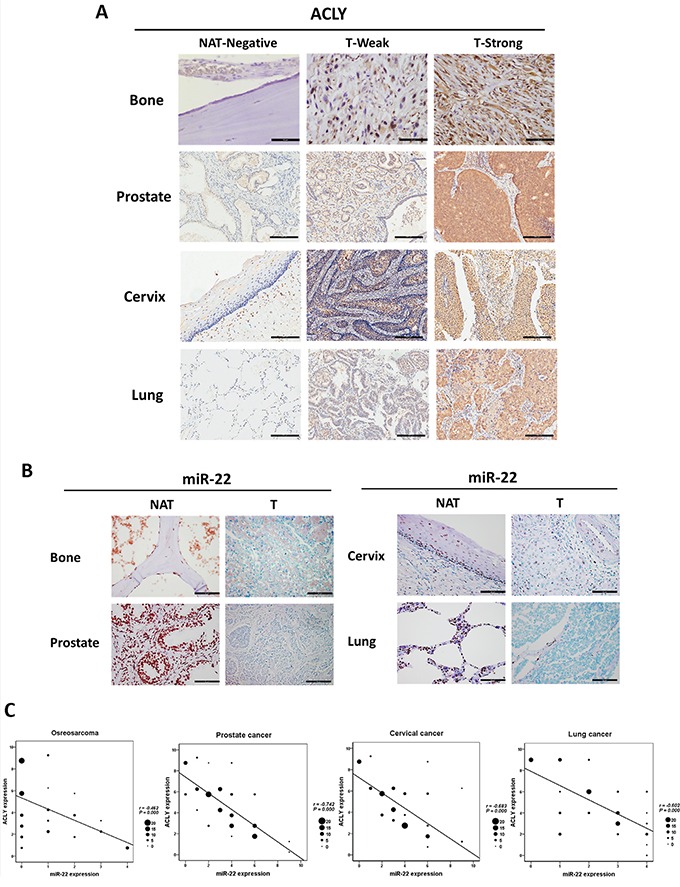
Verifications of the downregulation relationship between the ACLY protein and miR-22 in the clinical samples of osteosarcoma, prostate, cervical and lung cancers **A.** Immunohistochemistry against ACLY were performed between osteosarcoma (400X, scale bar 50 μm), prostate, cervical and lung cancers (100X, scale bar 200 μm) as well as the corresponding NAT samples. **B.** Representative images of miR-22 expression in osteosarcoma, prostate cancer, cervical cancer, lung cancer samples and the normal adjacent tissues displayed by RISH (400X, scale bar 50 μm). **C.** Analyses of the correlation relationship between ACLY and miR-22 expressions in the four tumors, and the size of the dot in the graphs was referred to the number of the cases.

For the further verification of the relationship between ACLY and miR-22 in osteosarcoma, prostate, cervical and lung cancers, immunohistochemistry (IHC) staining and a RISH method were performed on human tissue microarrays. Overall, the ACLY expression was predominantly located in the cytoplasm. Markedly elevated ACLY levels were uniformly observed across the four types of tumor tissues, yet the positive staining was hardly found in normal adjacent tissue (NAT) specimens (Figures 3A and Supplementary Figure S3). To detect miR-22 expression, based on the previous study [24], the normal and malignant colon tissues were determined respectively as the positive and negative control (Figure S3). As a result, fewer miR-22 staining was revealed in the four tumors than in the normal samples (Figures 3B and Supplementary Figure S3). The correlation analysis showed that the level of miR-22 was inversely associated with the ACLY protein expression among the four clinical samples respectively (Figure 3C).

### miR-22 suppresses *in vivo* tumor colonization and metastasis

**Figure 4:**
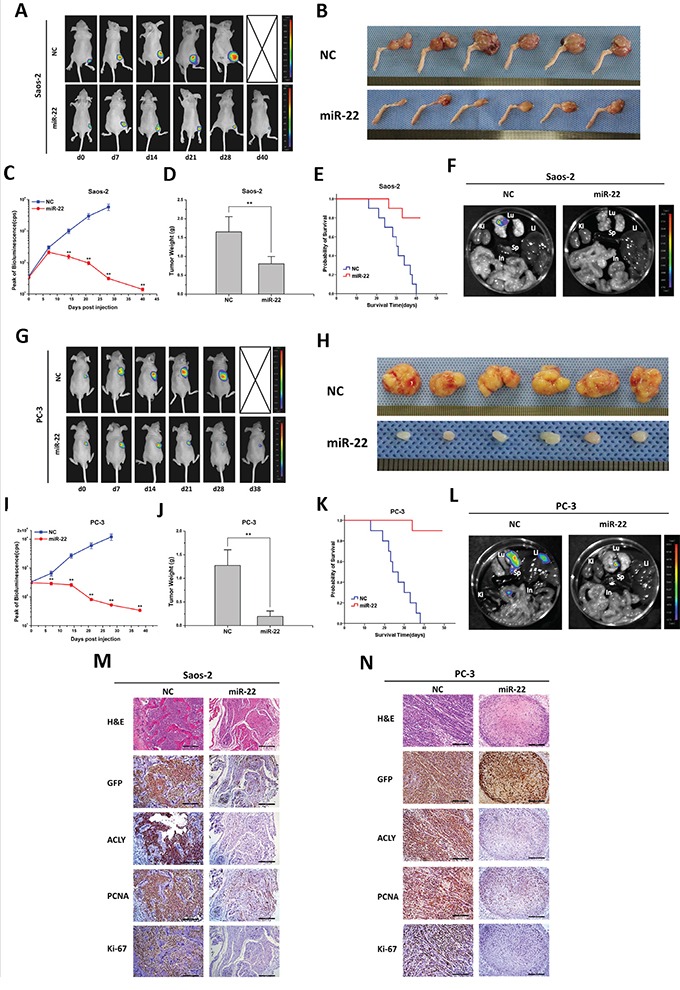
miR-22 suppresses tumor growth and metastasis in animal models of osteosarcoma and prostate cancer **A** and **G.** Representative bioluminescence images of mice with the orthotopic osteosarcoma model and xenografts of prostate cancer. Animals were imaged and treated with an intratumoral injection of miR-22 or NC miRNA every week until the date of death. **C** and **I.** Peak of bioluminescence (CPS) of the NC and miR-22 treatment groups were compared in the tumor-bearing mice (*P<0.05, **P<0.01). When the mice died or got sacrificed, the tumors were excised, photographed **B** and **H.** and weighed **D** and **J.** (**P<0.01), and the mice organs (Lu, Lung; Li, Liver; Sp, Spleen; Ki, Kidney; In, Intestine) were taken out for bioluminescence imaging **F** and **L.** The survival time of the mice with treatments of NC or miR-22 were analyzed by a Kaplan-Meier curve **E** and **K.** Finally, the tumor tissues were examined by H&E or IHC staining (100X, scale bar 200 μm) **M** and **N**.

We established the *in vivo* models of tumorigenesis on nude mice, and investigated the treatment effects of miR-22. For the Saos-2 originated tumor, in comparison with the NC-treated group, the therapeutic effect of miR-22 began to show 14 days after the drug administration, which followed by a continuous decline in the peak signal of fluorescence (Figures 4A and 4C). Evaluated by the mean tumor weight, the orthotopic tumors of the miR-22 treatment group weighed extremely lighter than those of the NC group (*P*=0.000; Figures 4B and 4D). No mice treated by the NC miRNA survived at the end of the experiment, whereas no distant metastasis was found in the miR-22 group (Figures 4E, 4F and Supplementary Figure S4). When the mice died or were sacrificed, X-ray radiographic images of the affected limb were taken immediately. In the NC group, high-density lesions were visualized in the metaphysis of the tibia, with evident periosteal reaction and bone destruction. By contrast, the mice treated with miR-22 only exhibited slight impairments in the cortex of the tibia (Figure S4). Afterwards, the orthotopic tumor was excised, and the tissues were subsequently fixed, embedded, sectioned and stained with hematoxylin & eosin (H&E). Microscopically, the tumors treated with NC miRNA displayed a disorderly growth pattern, and the normal substance of bone was extensively gnawed and invaded by the aggressive malignant cells. In contrast, after the treatment of miR-22, the tumor tissues developed sparse and well-differentiated structures. To confirm that the osteosarcomas were derived from previously injected Saos-2-GFP/luciferase cells, all tumor tissues were firstly stained against the GFP antibody using the IHC staining. The miR-22 treated tumors presented a distinct loss of ACLY protein expression in comparison with the NC group, with ensuing the decreasing expression of two classical proliferative markers, PCNA and Ki-67 (Figure 4M).

**Figure 5:**
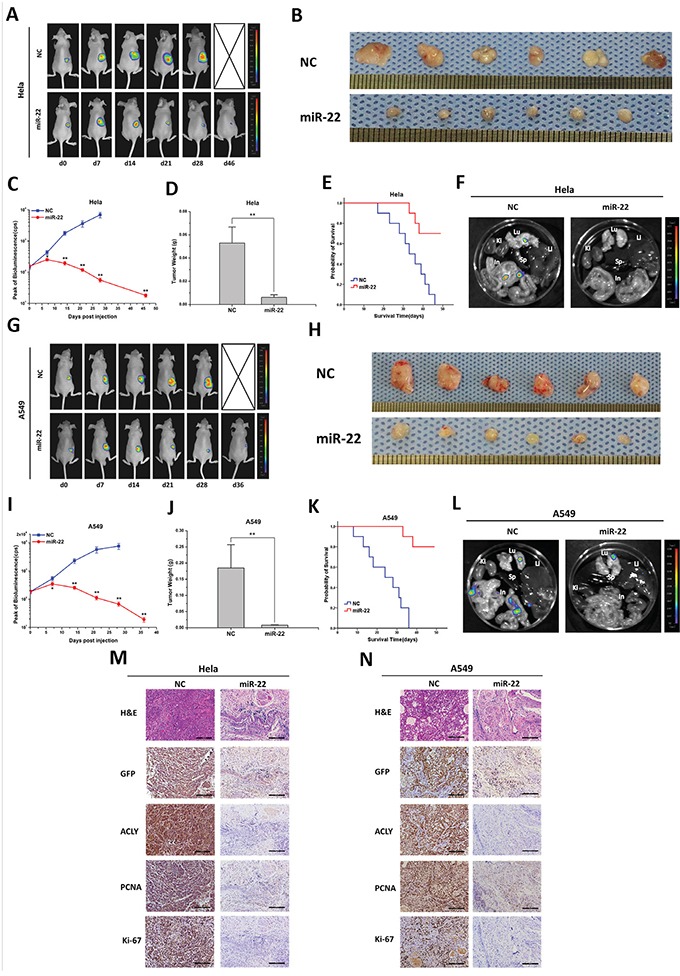
miR-22 suppresses tumor growth and metastasis in animal models of cervical and lung cancers **A** and **G.** Representative bioluminescence images of mice with the xenografts of cervical and lung cancers. **C** and **I.** Peak of bioluminescence (CPS) of the NC and miR-22 treatment groups were compared in the tumor-bearing mice (*P<0.05, **P<0.01). **B** and **H.** Typical photographs of the *ex-vivo* tumors. **D** and **J.** Statistical graphs of the tumor weights. **E** and **K.** The survival time curvesbetween the NC and miR-22 treatment groups. **F** and **L.** Representative bioluminescence images of the mice organs examined for metastatic probabilities. **M** and **N** Typical H&E and IHC staining images of the two tumor tissues (100X, scale bar 200 μm).

In the subcutaneously implanted models of PC-3 cells, the anti-tumor effects were seen with statistical differences 7 days after the drug injection (Figures 4G and 4I). The tumor weight, the distant organ metastasis manner and the survival time of the xenografts-bearing mice were found as the same trend as the Saos-2-injected mice (Figures 4H, 4J-4L and S4). Judging by H&E staining, compared to the miR-22 treatment group, the NC-treated tumors displayed dark-stained nuclei and twisty cord-like malignant phenotypes. Similar protein expression patterns of ACLY, PCNA and Ki-67 were revealed in the GFP positive tumor tissues by IHC staining (Figure 4N). Parallel findings were investigated in the mice models of Hela or A549 cells (Figures 5A-5N and S4).

### miR-22 downregulates ACLY-mediated *de novo* lipid synthesis

**Figure 6:**
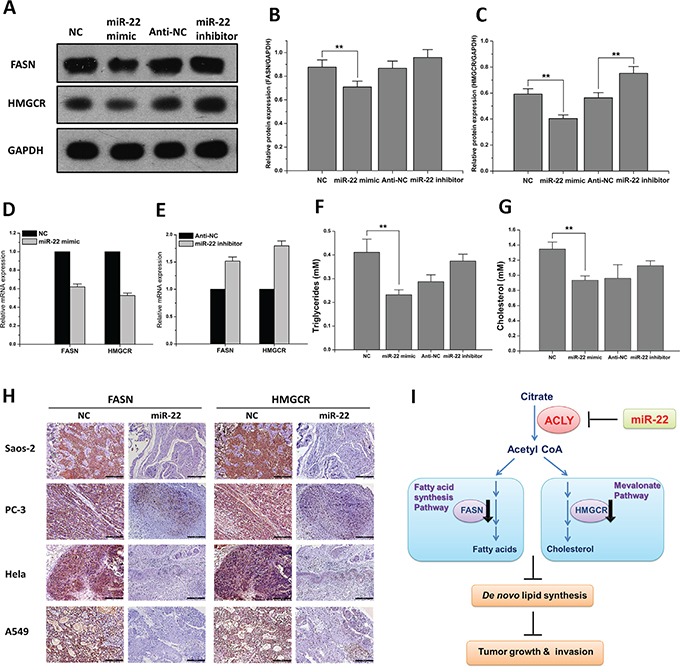
miR-22 reduces ACLY-mediated *de novo* lipid synthesis **A.** The protein expressions of FASN and HMGCR in the A549 cells transfected with the miR-22 mimic or inhibitor. **B** and **C.** Data presented the relative expressions of FASN and HMGCR protein levels (standardized to the intensity of GAPDH) (**P<0.01). **D** and **E.** The mRNA expressions of FASN and HMGCR in the A549 cells that were transfected as above. **F** and **G.** Analyses of the intracellular triglycerides and cholesterol levels after the treatment of miR-22 (**P<0.01). **H.** Representative IHC staining pictures of FASN and HMGCR expressions between the miR-22 and NC treatment groups of the four tumorigenic animal models (100X, scale bar 200 μm). **I.** Hypothesis of the miR-22-ACLY axis suppressing tumor growth and metastasis through *de novo* lipogenesis repression.

Fatty acid synthase (FASN) and 3-hydroxy-3-methylglutaryl-coenzyme A reductase (HMGCR) are the two crucial enzymes, respectively catalyzing the downstream lipid anabolism pathways for generating saturated fatty acids and cholesterol. Using the A549 cell as an *in vitro* experimental model, our results showed a concurrent downregulation both in the mRNA and protein levels of these two enzymes, which was in lines with the decreasing expression of ACLY treated by miR-22 (Figures 6A-6E). In the mice models of the four tumors, consistent with the decline of ACLY, evident lower levels of FASN and HMGCR expression were revealed by IHC staining after the anti-tumor treatment of miR-22 (Figures 6H and S5).

Fatty acids can be used as essential building blocks for the synthesis of triglycerides, which are mainly used for energy storage. Measured by the specific enzymatic assay kits, we found that in the A549 cells, the treatment of miR-22 mimic produced an inhibitory effect both in the cellular triglycerides and cholesterol levels, whereas the introduction of miR-22 inhibitor showed the opposite effect (Figures 6F and 6G). Triglycerides and cholesterylesters are stored in highly ordered cellular organelles--lipid droplets (LDs). To some extent, the number of intracellular LDs can reflect the reserve and accumulation of the lipid products. Oil red staining is a classic method in detecting LDs, and our results found that the introduction of miR-22 mimic triggered a notable decrease of lipid storage in the A549 cells compared to the NC-treated group. Similar inhibitory effects of miR-22-ACLY regulation were found among the Saos-2, PC-3 and Hela cell lines (Figure S5).

Taken together, we proposed a working model of a miR-22-ACLY axis that contributed to the suppression of *de novo* lipogenesis as well as the dysfunction of tumor growth and invasion (Figure 6I).

## DISCUSSION

Cancer is a highly pernicious disease where cells have lost their normal controls on cell growth and development. One of the most important metabolic hallmarks of cancer is increased *de novo* lipid synthesis [8]. *De novo* lipogenesis in the adult organism occurs mainly in the liver, adipose tissue and the lactating breast [25]. The carbon source essential for the lipid biosynthesis comes from cytosolic citrate that mainly originated from glucose. Catalyzed by the key enzyme ACLY, citrate is converted into acetyl-CoA that then enters into fatty acid synthesis and the mevalonate pathway. ACLY is a cytosolic enzyme that is substantially required for embryonic development [26]. It has been noted that neoplastic cells are able to reactivate *de novo* lipid synthesis in a manner similar to embryonic tissues [25]. The upregulated of ACLY has been reported as one of the common features during this metabolic remodeling, which contributes to tumor formation, proliferation and survival [27, 28]. And its inhibition turns out to become a potential approach in the cancer therapeutics.

MiRNAs are endogenous small non-coding RNAs, ~22 nt in length, that act as posttranscriptional regulators of gene expression [29]. And miRNAs fine-tune the activity of their targets involved in cancer metabolism [23]. Ectopic expressions of these miRNAs directly or indirectly mediate metabolic reprogramming. In this study, we have identified a novel link between miR-22, lipid anabolism and tumor suppression via downregulating the key metabolic enzyme ACLY. Some of the previous studies also reported a tumor-suppressive role of miR-22 in various neoplasms, including hepatocellular carcinoma [30], colon [24], ovarian [31] and breast cancers [32–34]. By aiming at a variety of targets in different tissues, miR-22 has been proved to reduce cancer cell proliferation, promote apoptosis as well as decrease cell migration and invasion. However, a recent study of the role of miR-22 in breast cancer proposed contradictory findings. Song *et al.* [35] have emphasized that miR-22 can enhance epithelial-mesenchymal transition and metastasis in mouse xenografts by silencing antimetastatic miR-200 through direct targeting of the TET family of methylcytosine dioxygenases, thus resulting in the inhibition of the miR-200 promoter demethylation. Accordingly, specific miRNAs like miR-22 may act mainly as a tumor suppressor whereas sometimes as an oncogene [36], depending on the different cellular environment, the diverse tumor systems and the particular functions of their target genes.

The study of Tomasetti *et al.* [37] on mesothelioma has discussed that ACLY could be indirectly inhibited by miR-126, which also led to the tumor suppression effect, whereas they tend to focus more on the interrelated impairment of mitochondrial respiration. In our work, we documented the direct downregulation relationship between miR-22 and ACLY in cancers such as osteosarcoma, prostate, cervical and lung cancers. The lipid metabolic reprogramming triggered by the miR-22 restoration and ACLY repression is associated with the inhibition of tumor development effects, resulting in the suppression of tumor growth and metastasis in the four types of cultured cancer cells as well as the animal models.

Lipids form a diverse group of water-insoluble molecules including triglycerides, phosphoglycerides, sterols and sphingolipids. Fatty acids are required for the production of phosphoglycerides, which, together with cholesterol, constitute the major structural components of cell membranes. Lipid droplets are also a repository of the membrane building blocks, such as phospholipids and sterols [38]. Cancer cells with high proliferating rates require large amounts of lipids as structural elements for biological membrane synthesis. In various types of tumors, regardless of the concentration of extracellular lipids, fatty acids are chiefly synthesized *de novo* [39]. Our data have shown that the inhibition of ACLY by miR-22 led to the decreasing expression of the downstream enzymes such as FASN and HMGCR, and thus ended up with the reducing output of triglycerides (the reservoir of fatty acids) and cholesterol. This, together with the effect of pro-apoptosis, contributed to the suppression of cancer cell growth.

The mevalonate pathway is another important branch within *de novo* lipid anabolism that facilitates the synthesis of cholesterol. Additionally, this biosynthesis pathway also generates essential intermediates required for the prenylation of small GTPases, including the farnesylation of Ras and the geranylgeranylation of Rho [40]. These lipid posttranslational modifications play significant roles in supporting the ability of Ras and Rho proteins to induce malignant transformation, invasion, and metastasis [41]. The downregulated effect of ACLY mediated by miR-22 may decrease the common intermediates expressions in the mevalonate pathway, and therefore lead to the suppression of tumor cell invasion and metastasis. In addition, the study of Zaytseva *et al.* [42] has stated that the inhibition of ACLY and FASN triggered the inactivation of a series of essential genes responsible for cell migration and invasion. Accordingly, miR-22-ACLY axis may have the similar effect on cancer cell mobility and the overall malignant progression.

It is known that the cancer metabolism is a complicated and precise network. MiRNAs regulate metabolic processes in a manner of fine-tuning that sometimes may produce extensive downregulating effects rather than simply blocking the specific target. In a related systemic study, Koufaris *et al.* [43] have revealed that in breast cancer there existed three target genes of miR-22 including ACLY, which participate in different metabolic pathways such as *de novo* lipogenesis, fatty acid elongation and mitochondrial one-carbon metabolism. And these targets were associated with poorer clinical outcomes that can be rescued by miR-22 modulation. Early on, Chen *et al.* [44] reported that also in breast cancer the important portal protein of glucose uptake, Glucose transporter 1 (GLUT1) was proved to be a direct target of miR-22, and dysregulated miR-22 expression functioned at the suppression of cancer cell proliferation and invasion. In this study, we chose four different types of cancer that are tightly associated with human life and health, and demonstrated that ACLY acted as a common target of miR-22 despite of the tumor types. The impairment of ACLY expression by miR-22 with ensuing inefficient *de novo* lipid synthesis may help to hinder the cell proliferation and invasion of these tumors. Data from the animal studies also indicated the beneficial effects of miR-22-ACLY regulation on the clinical prognosis. To sum up, miR-22 is considered to be a feasible approach in the suppression of various tumors, and ACLY is one of the most important component parts of its metabolic posttranscriptional control. Further work will be needed to explore and develop the clinical translational value of miR-22-ACLY axis.

## MATERIALS AND METHODS

### Clinical samples and immunohistochemistry

Cancer and adjacent noncancerous tissue microarrays of osteosarcoma (n =117), and prostate (n =135), cervical (n =129) and lung cancers (n =150) were obtained from US Biomax Inc. and Shanghai Outdo Biotech Co. Ltd. All of the tissue-chips were previously formalin-fixed, paraffin-embedded (FFPE) and sectioned into 5 μm thick slides. IHC staining was carried out with the primary antibody against ACLY (Abcam, Cambridge, MA, USA; 1:100). Secondary antibody staining and peroxidase detection were performed with an EnVision™ Detection Systems (Dako, Glostrup, Denmark) according to manufacturer's instructions. The staining results were evaluated and blindly ranked by two independent investigators using a grading system based on the percentage of positive tumor cells and the density of immunostaining. The extent score ranged from 0 to 3 (0, no positive cells; 1, < 10%; 2, 10%-50% ; 3, positive cells > 50%), and the intensity score was also measured on a scale of 0 to 3 (0, none; 1, weak; 2, moderate; 3, strong). The multiplication of the two scores yielded the final assessment of the ACLY protein levels, with grading from 0 to 9: negative (score 0-1), weak (score 2-4), strong (score 6-9).

### Cell lines

Human osteosarcoma (Saos-2), prostate cancer (PC-3), cervical cancer (Hela), lung cancer (A549), osteoblast (hFOB 1.19), normal prostate (RWPE-1), and normal lung (MRC-5) cells were purchased from the Cell Bank of the Chinese Academy of Sciences (Shanghai, China). Human normal primary cervical epithelia (normal) cells were obtained from Dr. Liu (Renji Hospital, School of Medicine, Shanghai Jiao Tong University, Shanghai, China). To generate stable bioluminescent Saos-2, PC-3, Hela, and A549 cell lines, a lentivirus vector containing luciferase and green fluorescent protein (GFP) was ordered from Shanghai GenePharma Company. The cells were transduced with the virus in the presence of polybrene (5 μg/mL) for 24 hours and then selected based on positive GFP expression under an inverted fluorescence microscope. All of the cells except for the hFOB 1.19 cells were incubated and maintained at 37°C in humidified air with 5% CO_2_. The culture temperature of the hFOB 1.19 cells was exclusively 33.5°C. The RWPE-1 cells were cultured in Keratinocyte-SFM Kit (Gibco Invitrogen, Carlsbad, CA, USA), whereas the others were maintained in DMEM/F-12 media (Gibco) supplemented with 10% FBS (Gibco).

### *In situ* hybridization

To detect miR-22, the specific miRCURY LNA™ probe labeled with digoxigenin at both 5′ and 3′ end was ordered from Exiqon Life Sciences. RNA *in situ* hybridization (RISH) was conducted using the REMBRANDT^®^ Universal RISH & HRP Detection Kit (Panpath, Amsterdam, The Netherlands) according to the manufacturer's instructions. The concentration of probe hybridized to the FFPE tissue was determined using previously published standard methods [45, 46]. The semi-quantitative analysis of miR-22 staining results were evaluated using the same methods as ACLY.

### Plasmid construction, siRNA and miRNA

To generate the protein overexpression, the ACLY coding sequence was cloned and inserted into pFLAG-CMV™-4 (Sigma, Saint Louis, MO, USA). To construct the luciferase reporter plasmid, the ACLY 3′ UTR was amplified and fused into the *XhoI* and *NotI* sites of a dual luciferase vector psiCHECK-2, a generous gift offered by Dr. Li (Alpert Medical School/Rhode Island Hospital, Brown University, Providence, RI, USA). Site-directed mutation of the miR-22 targeting site of the 3′ UTR fragment was performed by using a QuikChange Lightning Site-Directed Mutagenesis Kit (Stratagene, La Jolla, CA, USA). The siRNA targeting ACLY, miRNA mimic, inhibitor and agomiR of miRNA-22, along with the negative controls (NC and anti-NC) were synthesized by GenePharma. All cell transfections were carried out using the Lipofectamine^®^ 2000 Transfection Reagent (Invitrogen, Carlsbad, CA, USA) according to the manufacturer's recommendations. Primer sequences are listed in Table S1.

### Luciferase reporter assay

For the luciferase reporter detection, Saos-2, PC-3, Hela and A549 cells were seeded in 24-well plates and were cotransfected with the reporter plasmid and the miRNA. Twenty-four hours after incubation, firefly and *Renilla* luciferase activities were measured by using the Dual-Luciferase Reporter Assay System (Promega, Madison, WI, USA). The firefly luciferase activity was normalized by the *Renilla* luciferase activity as an internal standard of transfection efficiency. All the experiments were repeated with triplicate samples.

### Quantitative real-time PCR

Total RNA was isolated and purified using an E.Z.N.A. total RNA kit (Omega Bio-Tek, Doraville, GA, USA). For the quantitative detection of miRNA or mRNA expression, first strand cDNA was reverse-transcribed and real-time PCR was carried out using a SYBR PrimeScript miRNA RT-PCR kit (Takara, Otsu, Japan) following the manufacturer's protocol. U6 small nuclear RNA or glyceraldehyde-3-phosphate dehydrogenase (GAPDH) was used as an internal control. The 2^−ΔΔCt^ method was applied to quantify the relative expression levels of miRNA or mRNA, where C_t_ refers to the comparative cycle number at which the fluorescence of each sample passes the fixed threshold. The primers are listed in Table S1.

### Western blot

Cancers cells were harvested and extracted with ice-cold RIPA lysis buffer (Solarbio, Beijing, China), supplemented with a protease inhibitor. Proteins were separated by 10% SDS-PAGE, transferred to the PVDF membrane and probed with antibodies against ACLY (Abcam, 1:1000), FASN (Proteintech, Chicago, IL, USA; 1:200), HMGCR (Abcam, 1:1000) and GAPDH (Cell signaling technology, Danvers, MA, USA; 1:3000). A horseradish peroxidase-conjugated secondary antibody (Santa Cruz, Santa Cruz, CA, USA; 1:20000) was incubated and protein expressions were revealed by ECL detection reagents (Millipore, Billerica, MA, USA). The intensity of target protein bands was standardized to the intensity of the GAPDH bands.

### Cell proliferation assay

Cell proliferative activity was examined with a methyl thiazolyl tetrazolium (MTT) (Beyotime, Shanghai, China) assay. The cells were seeded in 96-well plates at a density of 5×10^3^ cells per well in 100 μL of growth medium. Twenty-four hours later, the cells were transfected with the miR-22 mimic, miR-22 inhibitor, NC, anti-NC and si-ACLY or ACLY-overexpressed vector separately cultured for another 24 h, 48 h or 72 h. Then, 10 μL of MTT (5 mg/mL) was added to each well. After a 4 h incubation at 37°C, the cells were disrupted in 100 mL of Dimethyl sulfoxide (DMSO), and the absorbance was measured at 490 nm on a microplate reader.

### Cell transwell assay

Cancer cells transfected with the above miR-22 analogs for 24h were harvested with 0.25% trypsin (Gibco), resuspended in serum-depleted media at a density of 1×10^5^ cells and plated onto the 8 μm invasion compartment (Corning, Corning, NY, USA) coated with basement membrane Matrigel (BD, San Jose, CA, USA). The chambers were placed into 24-well plates with media containing 10% FBS. After 24 hours of incubation at 37°C, the invasive cells on the lower membrane surface were fixed with formalin and stained with hematoxylin. The stained cells were imaged and counted by an inverted phase-contrast microscopy.

### Cell apoptosis

Flow cytometry analysis was performed to identify and calculate the percentage of apoptotic cells by using the Annexin V, FITC Apoptosis Detection Kit (Dojindo, Tokyo, Japan) following the manufacturer's guidelines.

### *In vivo* animal studies and miRNA treatment

BALB/c nu/nu mice at 3-5 weeks of age were provided and housed in the Laboratory Animal Center of Shanghai Institutes for Biological Sciences. Female mice were used for cervical cancer model studies and male mice for the other three types of cancer. All the experiments with live animals were approved by the Ethics Committee of Shanghai Jiao Tong University School of medicine and were conducted in compliance with the Guide for Care of Laboratory Animals as detailed by the National Ministry of Science. The mice were anaesthetized with chloral hydrate before any invasive operations. To establish the orthotopic osteosarcomas, GFP/luciferase-expressing Saos-2 cells (1×10^7^) were resuspended in 50 μL of PBS, aspirated into a 1 mL syringe fitted with a 25-gauge needle, and injected into the cortex of the anterior tuberosity of the right tibia. Fourteen days after implantation, the mice were randomly divided into two groups of 10 mice per group. The miR-22 group was intratumorally treated twice a week with the agomiR of miR-22 carried by the *in vivo*-jetPEI^®^ Delivery Reagent (Polyplus Transfection, New York, NY, USA) according to the manufacturer's protocols, and correspondingly the NC group was injected with the negative control (NC) miRNA. For the xenograft cancer models, stable PC-3 (2.5×10^6^), Hela (3×10^6^) and A549 (5×10^6^) cells transduced with GFP/luciferase lentivirus were resuspended in 50 μL of PBS, and inoculated subcutaneously into the right flanks of the nude mice. Seven days post injection, mice bearing the specific cancer were randomly assigned and treated intratumorally using the same method performed with the Saos-2 tumors. The effect of the miRNAs intervention was observed and imaged weekly by a NightOWL LB983 *in vivo* imaging system (Berthold, Bad Wildbad, Germany) from the first day of the drug administration until the day the mouse died. And the life span of the mice was also monitored. After the final imaging, all of the mice were sacrificed, and the organs (lung, liver, spleen, kidney and intestine) were removed and examined by bioluminescence imaging. The tumors were excised, photographed and weighed. The tumor tissues were fixed in 4% paraformaldehyde, embedded with paraffin and sectioned into 5 μm thick slides. IHC staining was subsequently performed to detect the protein expressions of GFP, ACLY, PCNA, Ki-67, FASN and HMGCR within the implants.

### Bone X-ray scan

The mice bearing with the orthotopic osteosarcoma were additionally imaged by X-ray radiography using a MX-20 Digital system (Faxitron, Tucson, Arizona, USA) at 20 kV for 20 sec immediately following the sacrifice by chloral hydrate anesthesia.

### Triglycerides and cholesterol assay and LDs staining

Intracellular triglycerides and cholesterol were estimated using enzymatic assay kits (Applygen Technologies, Beijing, China) according to the manufacturer's recommended protocol. Intracellular lipid droplets were detected by the oil red O reagent (Sigma) according to the manufacture's specification.

### Statistical analysis

All statistical analyses were conducted using the SPSS 17.0. All of the experiments were analyzed by Student's *t* test or Mann-Whitney U test. Spearman's rank correlation was used to determine the relationship between the miR-22 and ACLY expressions. The survival analysis of the mice was calculated using a Kaplan-Meier method. Data were presented as mean ± SD and *P*<0.05 was determined to be statistically significant.

## SUPPLEMENTARY FIGURES AND TABLES


