# Supplementary material for: miR-22 inhibits tumor growth and metastasis by targeting ATP citrate lyase: evidence in osteosarcoma, prostate cancer, cervical cancer and lung cancer
Source: Oncotarget. 2016 Jun 14;7(28):44252–65. doi: 10.18632/oncotarget.10020 (PMC5190093; doi:10.18632/oncotarget.10020)
Supplement: Supplementary file 1 [file oncotarget-07-44252-s001.pdf]

miR-22 inhibits tumor growth and metastasis by targeting ATP citrate lyase: evidence in osteosarcoma, prostate cancer, cervical cancer and lung cancer

SUPPLEMENTARY FIGURES AND TABLE

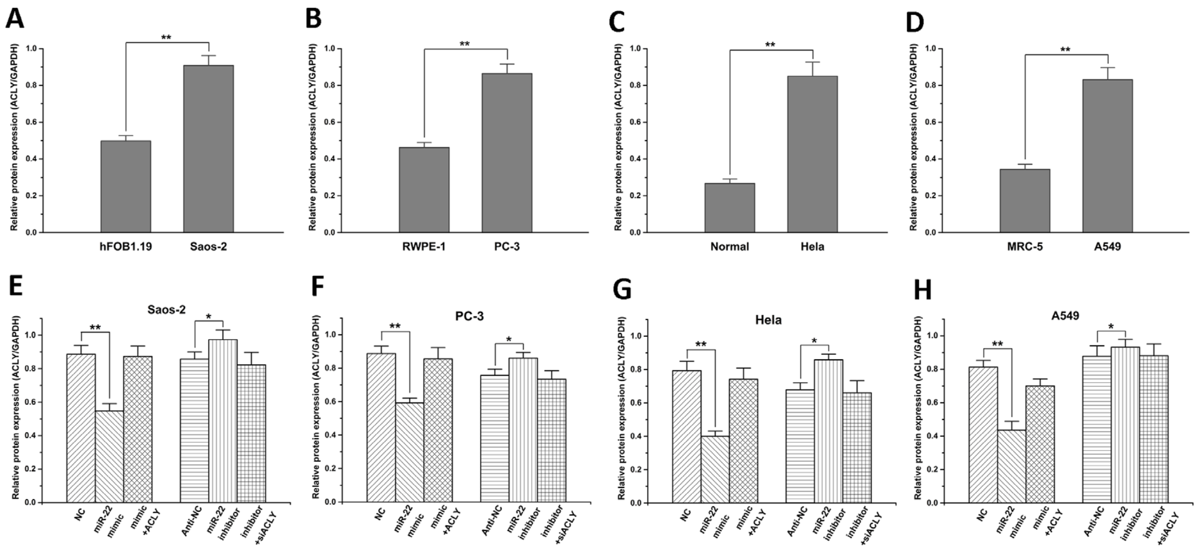

Supplementary Figure S1: A-D. Data represented the relative expression of ACLY protein levels (standardized to the intensity of GAPDH) in Saos-2, PC-3, Hela and A549 cells and their normal controls (**$P<0.01$). **E-H.** Data represented the relative expression of ACLY protein levels (standardized to the intensity of GAPDH) in the four cancer cell lines treated with miR-22 mimic or miR-22 inhibitor as well as cotransfected by ACLY-overexpressed vector or siRNA (*$P<0.05$, **$P<0.01$).

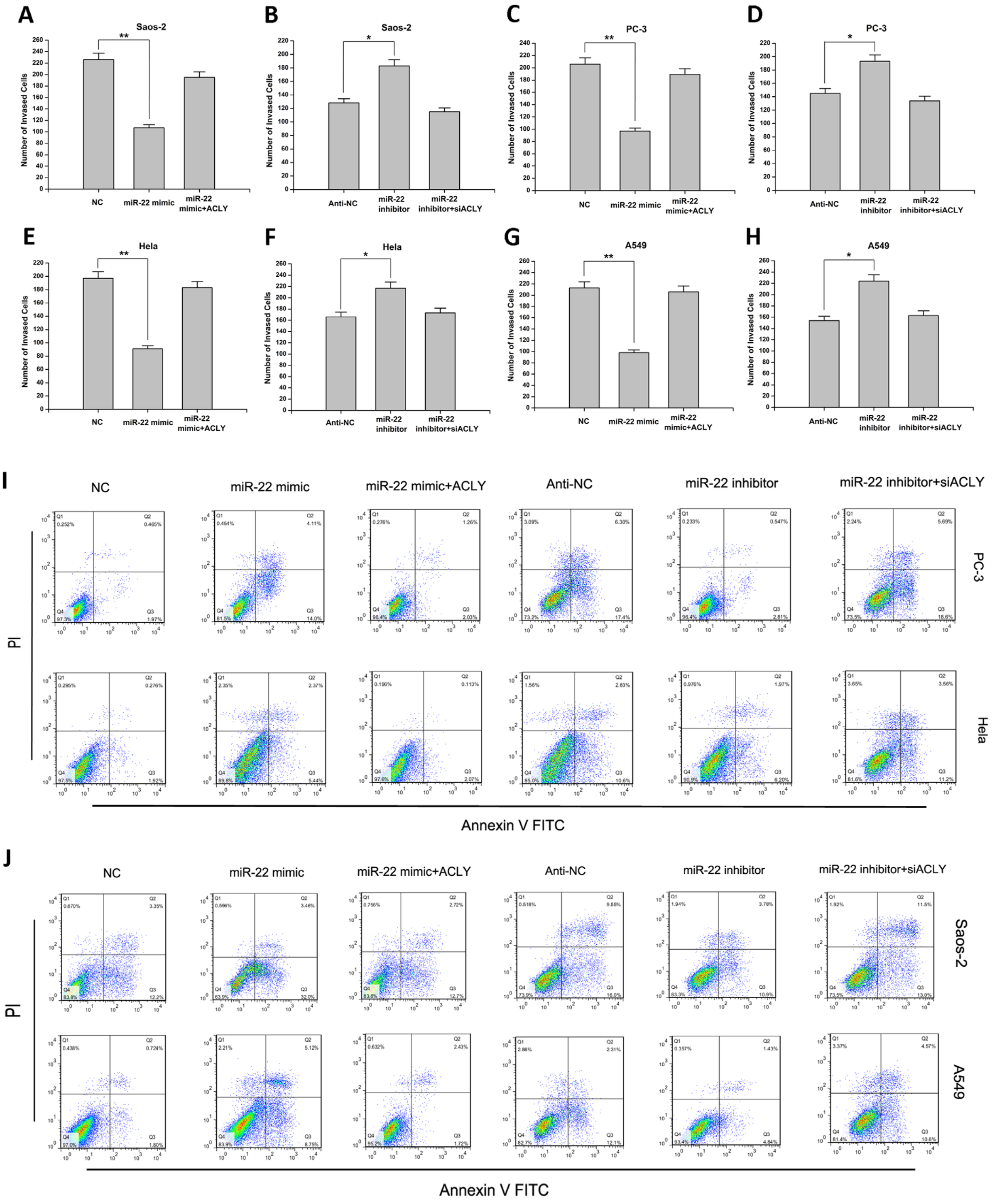

Supplementary Figure S2: A-H. Data represented the counted number of the invaded cells during the transwell assay (**P*<0.05, ***P*<0.01). **I** and **J.** Saos-2, PC-3, Hela and A549 cells were transfected with the miR-22 mimic or inhibitor, double-stained by Annexin V / PI and examined by flow cytometry for cell apoptosis.

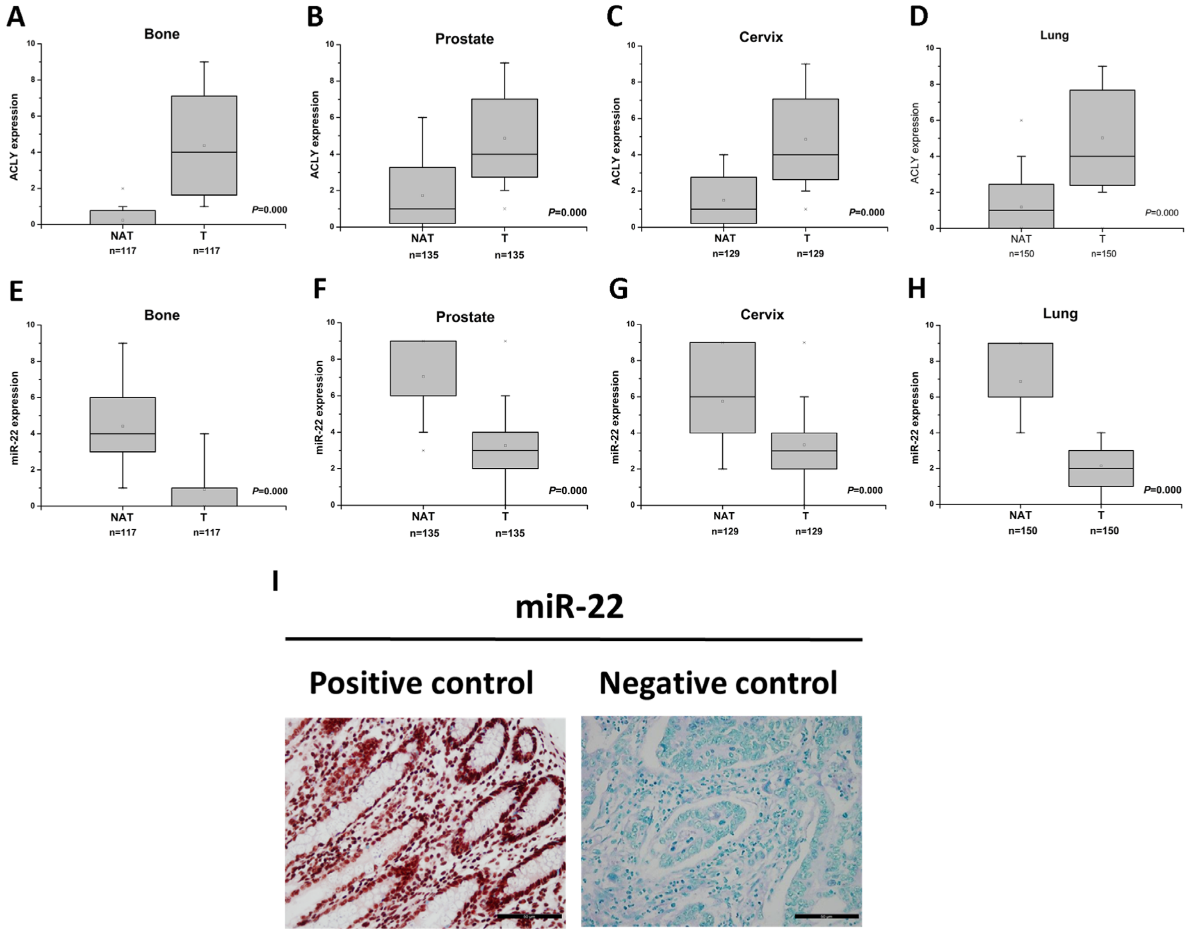

Supplementary Figure S3: A-D. Data represented the distribution of ACLY staining scores between the NAT samples and the tumor tissues (T). **E-H.** Data represented the distribution of the miR-22 expression between the NAT and T samples. **I.** Positive control and negative control stainings of miR-22 detected by RISH (400X, scale bar 50 μm).

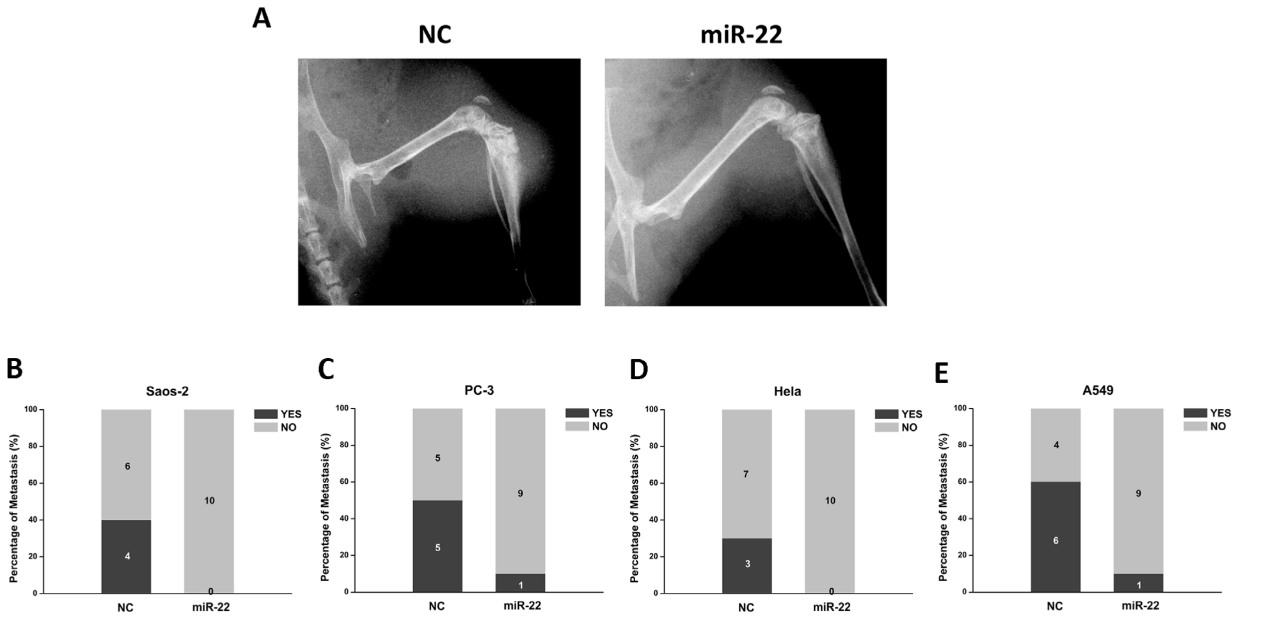

Supplementary Figure S4: A. Representative X-ray images of the *in vivo* orthotopic osteosarcoma model treated by NC or miR-22, separately. **B-E.** The percentages of distant metastasis after the different miRNA treatments in the four *in vivo* tumor models.

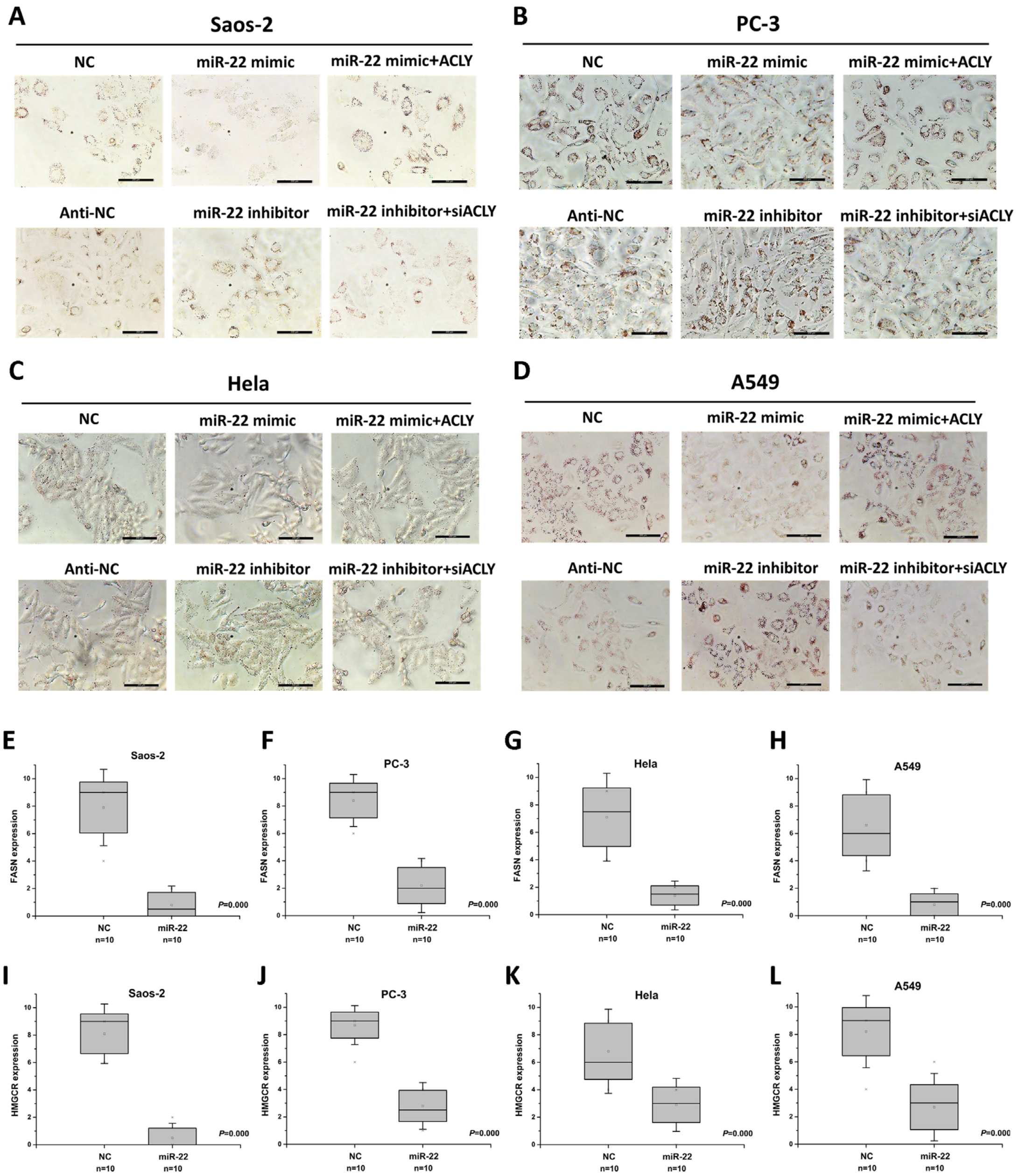

Supplementary Figure S5: A-D. Oil red staining pictures of Saos-2, PC-3, Hela and A549 cells transfected with the miR-22 mimic or inhibitor (200X, scale bar 100 μm). **E-L.** Data represented the distribution of FASN or HMGCR IHC staining scores of the mice tumor tissues between the NC and miR-22 groups.

Supplementary Table S1: Primer sequences used in real-time PCR and the plasmid construction; sequences of siRNA and agomiR fragments

**Primers for quantitative real-time PCR**		
	Sense Primer (5' to 3')	Antisense Primer (5' to 3')
miR-22	CCAGCTAAAGCTGC CAGTTGAAGAACTG	Uni-miR qPCR Primer supplied by the SYBR PrimeScript miRNA RT-PCR kit
miR-27a	CGTGTTCACAGTGGCTAAGTTCCGC	*Vide supra*
miR-27b	CGCTTTGTTCACA GTGGCTAAGTTCTGC	*Vide supra*
U6	CTCGCTTCGGCAGCACA	AACGCTTCACGAATTTGCGT
FASN	TATGAAGCCATCGTGGACGG	CATGCTGTAGCCCACGAGT
HMGCR	GCCCTCAGTTCCAACTCACA	TTCAAGCTGACGTACCCCTG
GAPDH	GAAACCAGATCTCCACCGCA	GCGCCCAATACGACCAAATC
**Primers for quantitative plasmid construction**		
	Sense Primer (5' to 3')	Antisense Primer (5' to 3')
ACLY	ATGTCGGCCAAGGCAATTTC (*HindIII*)	CATGCTCATGTGTTCCGGAA (*EcoRI*)
ACLY 3'UTR-WT	CAGAGCCAGGAA CCCTACTGCAGTAA (*Xhol*)	TTGTTACTATATAGTTTATTTAA ACCAGACTATGATAATACAGAGAAG (*NotI*)
ACLY 3'UTR-Mut	GTAATCCACAAAGATTCTG GCGTGCTGCCACCTCAGTCTCTTC	GAAGAGACTGAGGTGGCA GCACGCCAGAATCTTTGTGGATTAC
**siRNA and agomiR fragments**		
	Sense Primer (5' to 3')	Antisense Primer (5' to 3')
siACLY	GCGUGGAUGAGAAACUGAATT	UUCAGUUUCUCAUCCACGCTT
miR-22 agomiR	AAGCUGCCAGUUGAAGAACUGU	AGUUCUUCAACUGGCAGCUUUU
NC agomiR	UUCUCCGAACGUGUCACGUTT	ACGUGACACGUUCGGAGAATT
